# Ionic Liquid Microenvironment Engineering in HKUST-1 for Efficient Photothermal CO_2_ Cycloaddition

**DOI:** 10.3390/molecules31132332

**Published:** 2026-07-03

**Authors:** Renkun Huang, Haohao Yan, Runling Huang, Chen Zhou, Qiuzhong Li, Lu Chen, Ruowen Liang

**Affiliations:** 1 Province University Key Laboratory of Green Energy and Environment Catalysis, Ningde Normal University, Ningde 352100, China; t1432@ndnu.edu.cn (R.H.);; 2 Fujian Provincial Key Laboratory of Featured Materials in Biochemical Industry, Ningde Normal University, Ningde 352100, China; 3 State Key Laboratory of Photocatalysis on Energy and Environment, Fuzhou University, Fuzhou 350002, China

**Keywords:** metal–organic frameworks (MOFs), photocatalysis, ionic liquids, synergistic catalysis

## Abstract

A novel composite catalyst for photothermal CO_2_ cycloaddition was developed by integrating the ionic liquid 1-ethylpyridinium bromide (EPB) with a copper-based metal–organic framework (HKUST-1). HKUST-1 was synthesized via a hydrothermal method and functionalized with EPB through a wet impregnation strategy to enhance its catalytic performance. Under xenon lamp irradiation and optimized conditions (80 °C, 1 MPa CO_2_ pressure, 12 h, and 0.07% mol of TBAB bromide as a co-catalyst), the HK@EPB composite exhibited outstanding performance in catalyzing the conversion of CO_2_ and various epoxides into cyclic carbonates. The exceptional catalytic activity arises from a synergistic multicomponent mechanism: the incorporation of EPB not only enhances CO_2_ adsorption capacity but also provides photothermal energy for the reaction; simultaneously, EPB dissociates bromide ions to effectively initiate epoxide ring-opening. In particular, propylene oxide achieved a selectivity of 95% for the desired cyclic carbonate, surpassing most previously reported MOF-based catalysts. This system enables efficient catalysis under mild conditions through the synergistic contributions of the high CO_2_ adsorption capacity and Cu^2+^/Cu^+^ redox-mediated electron transfer of HKUST-1, the provision of nucleophilic Br-species from EPB to promote epoxide ring-opening, and the cooperative effect of TBAB. This study demonstrates that ionic-liquid-functionalized MOF composites can serve as sustainable and versatile catalytic platforms, offering an environmentally friendly pathway for large-scale CO_2_ utilization.

## 1. Introduction

Carbon dioxide (CO_2_), as an abundant, non-toxic, and renewable C1 resource, has attracted extensive attention in recent years due to its potential for conversion into value-added chemicals [1,2,3]. Among various CO_2_ utilization strategies, the cycloaddition of CO_2_ with epoxides to form cyclic carbonates represents one of the most promising routes because of its 100% atom economy and the high application value of the products [4,5,6,7,8,9].

In conventional thermal catalytic processes [10,11,12], Lewis acids activate epoxides, halide anions (Br^−^) perform nucleophilic ring-opening, and then CO_2_ inserts to form cyclic carbonates. However, this process requires high temperatures of 100–150 °C and high pressures of 1–2 MPa CO_2_ [13,14,15], and homogeneous co-catalysts suffer from separation difficulties and corrosion issues [16,17].

Photothermal catalytic CO_2_ cycloaddition, which utilizes solar energy to drive the reaction, has emerged as a promising alternative. Current systems mainly rely on noble metals or black MOF/carbon-based materials for photothermal conversion [18,19]. Nevertheless, three major challenges remain: most systems still require temperatures > 100 °C or external heating; the photochemical and photothermal effects are difficult to distinguish; and the immobilization of nucleophilic halide sources onto photoresponsive supports to construct dual-functional photothermal catalysts is still very limited [20,21].

Immobilizing ionic liquids onto photoactive MOFs can combine nucleophilic halides, CO_2_ adsorption, and Lewis acidity [22,23,24]. Current research indicates that nitrogen-rich poly(ionic liquid)s can efficiently catalyze the cycloaddition reaction of CO_2_ and epoxides under mild, solvent-free conditions, while also enabling effective catalyst recovery and reuse [25]. Particularly, several MOFs, such as UiO-66, MIL-101, and NH_2_-MIL-125, have been reported as effective supports for ionic liquid immobilization [26,27], combining both CO_2_-philicity and visible-light-driven photo-induced activation of CO_2_ [28,29,30,31]. Herein, we report a dual-functional system based on HKUST-1 functionalized with ethylpyridinium bromide (HK@EPB). In this system, HKUST-1 serves as a porous, photoresponsive Lewis acid framework, while EPB provides nucleophilic Br^−^. The two components synergistically drive the photothermal cycloaddition: copper paddlewheel sites activate epoxides; light-induced charge transfer generates localized heating to accelerate ring-opening; the microporous structure enriches CO_2_; and photogenerated electrons lower its activation barrier. The Br^−^ of EPB attacks the activated epoxide, while the pyridinium cation stabilizes intermediates and enriches substrates. By immobilizing EPB onto HKUST-1, Br^−^ is positioned adjacent to the copper sites, enabling an efficient tandem activation–nucleophilic attack process within a nanoconfined space. This system achieves true photothermal–nucleophilic synergy, rather than a simple superposition of photocatalysis and thermal catalysis. Characterization confirms the successful incorporation of EPB without compromising the structural integrity of HKUST-1, and the catalyst exhibits excellent activity, substrate compatibility, and recyclability.

## 2. Methodology

### 2.1. Materials and Chemicals

Copper nitrate trihydrate was provided by Aladdin Chemical Co., Ltd. (Tianjin, China). N,N-Dimethylformamide (DMF), anhydrous methanol (MeOH), and absolute ethanol (EtOH) were purchased from Sinopharm Chemical Reagent Co., Ltd. (Shanghai, China). 1,2-Butylene oxide, 1,2-epoxyhexane, 1-ethylpyridinium bromide hydrobromide, and propylene oxide were obtained from Shanghai Aladdin Reagent Co., Ltd. (Shanghai, China). Cyclohexene oxide and propylene oxide (methyloxirane) were provided by TCI Chemical Reagents (Shanghai, China).

### 2.2. Catalyst Preparation

#### 2.2.1. Synthesis of HKUST-1

**Figure 1:**
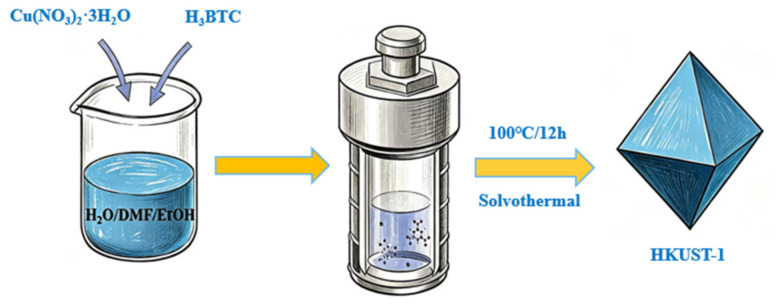
The synthetic image of HKUST-1.

A blend of 2.00 g Cu(NO_3_)_2_·3H_2_O (8.29 mmol) and 0.96 g 1,3,5-Benzenetricarboxylic acid (H_3_BTC) (4.57 mmol) was dissolved individually in 30 mL of a deionized water/DMF/ethanol solvent in a 1:1:1 (*v*/*v*/*v*) ratio. The solutions were agitated until complete dissolution and then merged to create a uniform blue solution. This mixture was then placed into a 100 mL Teflon-lined autoclave and heated at 100 °C for 12 h (Figure 1). After cooling to room temperature, the blue precipitate was collected by centrifugation, washed three times with ethanol, and dried under vacuum at 100 °C to obtain HKUST-1.

#### 2.2.2. Preparation of EPB-Functionalized HKUST-1

**Figure 2:**
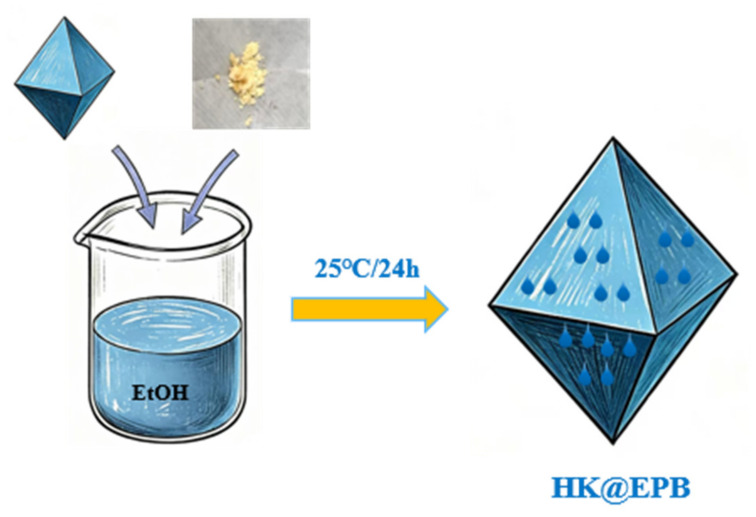
The synthetic image of HK@EPB.

Masses of 0.3 g of HKUST-1 and 0.6 g of EPB were accurately weighed and dispersed in 50 mL of ethanol, followed by 24 h of wet impregnation with continuous stirring(Figure 2). The resulting mixture was filtered, washed three times with ethanol, and then dried under vacuum at 100 °C overnight. The obtained material was further activated under vacuum at 150 °C for 5 h, ultimately yielding the HK@EPB composite.

### 2.3. Activity Test Conditions

CO_2_ cycloaddition reactions were carried out in a 100 mL high-pressure stainless-steel reactor (CEL-HPR100T, Beijing Zhongjiao Jinyuan Technology Co, Ltd., Beijing, China) fitted with a magnetic stirrer and temperature control system. A quartz optical window was installed directly above the reactor, with the Xe lamp light source horizontally aligned to the window for external irradiation, enabling efficient photocatalytic reaction while maintaining high-pressure sealing. The standard reaction mixture comprised 5 mL of propylene oxide (PO, 71.5 mmol), 80 mg of catalyst, and 0.07% mol of tetrabutylammonium bromide (TBAB). The reactor was pressurized with CO_2_ to 1 MPa. Photothermal catalysis was conducted at 80 °C under irradiation from a 300 W Xe lamp (≈200 mW/cm^2^, λ ≥ 420 nm, CEL-HXF300-T3, Beijing Zhongjiao Jinyuan Technology Co, Ltd.) for 12 h. Upon completion of the reaction, the catalyst was filtered, and the yield of propylene carbonate (PC) was determined using a Shimadzu GC-2012 gas chromatograph equipped with a capillary column and flame ionization detector (FID) (Shimadzu, Tokyo, Japan).

### 2.4. Catalyst Characterization

X-ray diffraction (XRD) was employed to determine the phase composition of the photocatalysts, using a Cu Kα X-ray source (λ = 0.15406 nm) on a Bruker D8 diffractometer (Bruker, Karlsruhe, Germany). The morphology and microstructure of the prepared photocatalysts were analyzed by scanning electron microscopy (ZEISS GeminiSEM 300) (Carl Zeiss AG, Oberkochen, Germany). The Brunauer–Emmett–Teller (BET) surface area was determined using an ASAP 2020 analyzer (Micromeritics Instrument Corporation, Norcross, GA, USA) via the multipoint BET method. The surface chemical states of elements in the samples were investigated by X-ray photoelectron spectroscopy (XPS, VG ESCALAB 250) (Thermo Fisher Scientific, East Grinstead, UK) with a monochromated Al-Kα X-ray source. UV-Vis absorption spectra of the photocatalysts were recorded on a Cary 500 spectrophotometer (Agilent Technologies, Santa Clara, CA, USA) using BaSO_4_ as a reflectance standard. Photoluminescence (PL) spectra were measured at room temperature using a fluorescence spectrophotometer (FLS 980) (Edinburgh Instruments, Livingston, UK) with an excitation wavelength of 400 nm. The thermal stability and composition of the samples were preliminarily assessed by thermogravimetric analysis (TGA) on a TA TGA55 thermal analyzer (TA Instruments, New Castle, DE, USA).

### 2.5. Photoelectrochemical Measurements

The AutolabPGSTA204 electrochemical system (Metrohm Autolab, Utrecht, The Netherlands) was used to measure photocurrent response and electrochemical impedance spectroscopy (EIS) with a standard three-electrode cell. To prepare the working electrode, the following process was followed: First, 10 mg of photocatalyst powder was added to 1 mL of DMF to create a slurry. This slurry was then spread onto the conductive surface of FTO glass, and allowed to dry in air. The reference electrode and the counter electrode used in the experiment were Ag/AgCl and Pt wire, respectively. The photocurrent was measured with a Xenon lamp (300 W) (Beijing Perfectlight Technology Co., Ltd., Beijing, China) switched on and off under a constant bias of 0.5 V. A 0.2 M Na_2_SO_4_ (containing 0.1 M Na_2_SO_3_/Na_2_S) mixture solution was used as the electrolyte. The electrochemical impedance spectroscopy (EIS) test was performed over a frequency range from 0.01 to 105 under open circuit potential. A 5 mM K_3_[Fe(CN)_6_]/K_4_[Fe(CN)_6_] solution was used as the electrolyte.

## 3. Results

**Figure 3:**
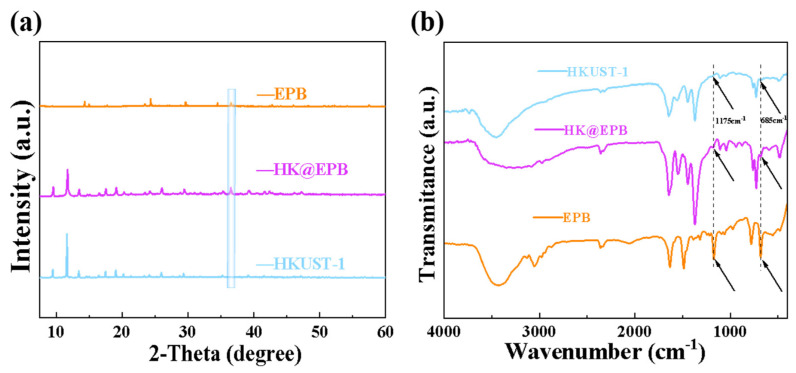
(**a**) XRD patterns, (**b**) FT-IR spectra of HKUST-1 and HK@EPB.

The analysis in Figure 3a confirmed that the diffraction peaks of pristine HKUST-1 matched well with the standard patterns reported in the literature [32,33]. Notably, the crystal structure and phase integrity of the composite remained intact after EPB immobilization, suggesting that the incorporation of the ionic liquid did not compromise the structural stability of the HKUST-1 framework. Furthermore, the XRD pattern of the EPB-immobilized composite exhibited a distinct peak at approximately 37°, which was absent in the pristine HKUST-1. This new peak is attributed to the characteristic diffraction of the EPB, providing clear evidence of its successful integration into the composite.

Both pristine HKUST-1 and the EPB-immobilized composite, as shown in Figure 3b, displayed distinct FT-IR absorption bands at 1646, 1447, 1378, 729, and 476 cm^−1^, consistent with previously reported spectral characteristics [34]. The peaks at 1646 cm^−1^ and 1447 cm^−1^ correspond to the asymmetric and symmetric stretching vibrations of the carboxylate group (-O-C-O-), respectively. The absorption band at 1378 cm^−1^ is attributed to C-O related vibrations associated with the carboxylate groups. Additionally, after EPB immobilization, the bands at 729 cm^−1^ and 476 cm^−1^ corresponding to Cu-O bending and stretching vibrations remain visible, confirming the structural integrity of the HKUST-1 framework. Significantly, novel vibrational features were identified in the composite spectrum: the vibration of the pyridine ring skeleton was observed at 1175 cm^−1^, and the out-of-plane bending vibration peak of the pyridine ring C-H is detected at 685 cm^−1^, both characteristic of EPB.

**Figure 4:**
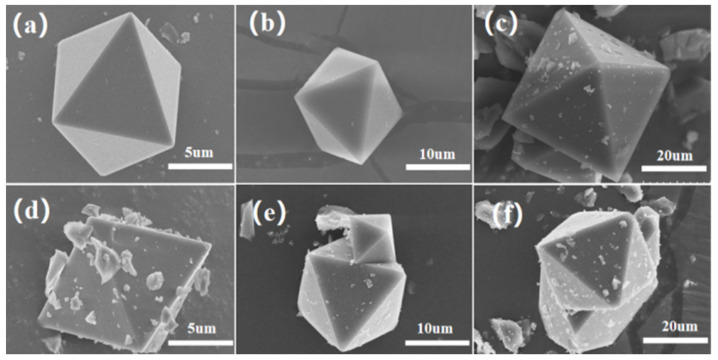
(**a**,**b**) SEM image of HKUST-1, (**c**–**f**) SEM image of HK@EPB.

Morphological and mechanistic insights from scanning electron microscopy analysis (SEM): The pristine HKUST-1 material, as shown in Figure 4a,b, exhibits a distinct octahedral morphology with smooth surfaces, featuring an average particle size of approximately 5 μm [35]. Upon immobilization of EPB, rice-like deposits appear on the initially smooth facets of the octahedrons, as shown in Figure 4c–f.

**Figure 5:**
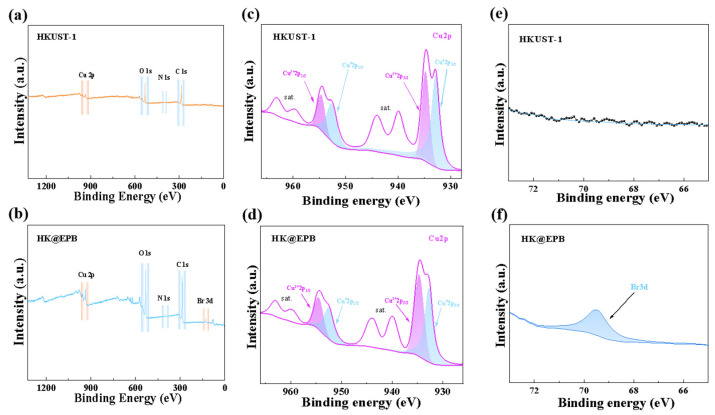
The XPS (**a**,**b**) survey spectra, (**c**,**d**) Cu 2p, (**e**,**f**) Br 3d of samples.

XPS analysis revealed that the HKUST-1 framework consists of Cu, O, C, and N elements, while the EPB composite additionally exhibited the presence of Br signals (Figure 5a,b), confirming the successful immobilization of EPB. The Cu 2p spectrum of HKUST-1 (Figure 5c,d) showed dominant peaks at 933.8 eV and 953.3 eV, which are attributed to monovalent copper (Cu^+^), along with weaker peaks at 935.4 eV and 955.3 eV, assigned to divalent copper (Cu^2+^) [36]. The accompanying shake-up satellite peaks further verified the coexistence of mixed Cu^+^/Cu^2+^ valence states. In contrast to pristine HKUST-1, the composite material (Figure 5e,f) exhibited distinct Br^−^ signals originating from the EPB ionic liquid.

To quantify the loading amount of EPB in the composite, we performed ion chromatography to measure the bromine content in the composite. As shown in Table 1, the test results show that the bromine content in the composite is 1595.110 mg/kg, and all of the bromine in the composite originates entirely from EPB.

**Figure 6:**
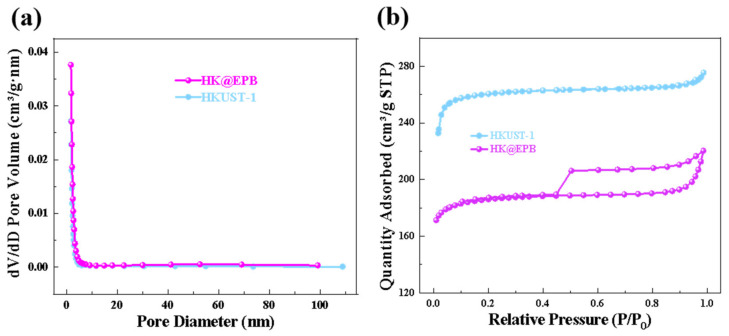
(**a**) The pore size distribution of HKUST-1 and HK@EPB, (**b**) N_2_ adsorption and desorption isotherms.

The porosity and specific surface area of the samples were assessed using N_2_ adsorption–desorption measurements. The pristine HKUST-1 displayed a BET surface area of 1088 m^2^/g, which decreased notably to 558 m^2^/g after immobilization of the ionic liquid, representing nearly a twofold reduction, as shown in Table 2 and Figure 6a. Concurrently, the pore volume of the composite also decreased after EPB incorporation. These reductions are attributed to steric hindrance imposed by the confined ionic liquid within the HKUST-1 framework.

**Figure 7:**
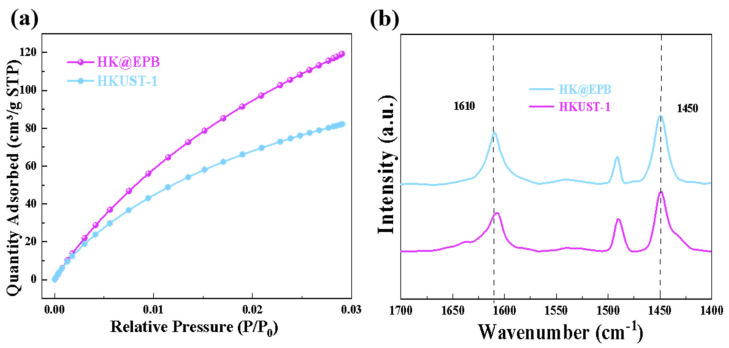
(**a**) Carbon dioxide adsorption and (**b**) pyridine infrared curves of HKUST-1 and HK@EPB.

Compared with pristine HKUST-1, the HK@EPB composite exhibits significantly enhanced Lewis acidity and CO_2_ adsorption capacity, as evidenced by the CO_2_ adsorption isotherms (Figure 7a) and pyridine-adsorbed FT-IR spectra (Figure 7b). Notably, the pristine HKUST-1 shows a Lewis acid site density of 39.74 μmol·g^−1^, whereas the incorporation of EPB substantially increases this value to 56.57 μmol·g^−1^ in the composite. This increase may be attributed to the composite preparation process (e.g., washing steps), which helps expose more metal active sites. Although the ionic liquid itself does not possess Lewis acidity, the post-treatment procedures such as washing employed during the loading of EPB onto HKUST-1 may promote the effective exposure of metal sites within the MOF pores, thereby increasing the overall Lewis acid site density of the composite. Meanwhile, related studies have demonstrated that nitrogen-containing porous organic polymers hold significant potential for applications in the field of carbon dioxide capture, utilization, and storage (CCUS) [37].

The Lewis acid site concentrations were calculated using the methodology described by Emeis et al. [38]. Generally, the characteristic band at around 1450 cm^−1^ in the pyridine-adsorbed FT-IR spectrum is attributed to the vibration of pyridine coordinated to Lewis acid sites. However, in MOF materials such as HKUST-1, these signals may overlap with the stronger bands in the 1400–1600 cm^−1^ region [39,40,41], which originate from the vibrations of Cu-carboxylate linkages and aromatic rings [42]. Therefore, additional signals in the 1400–1600 cm^−1^ range were also considered as auxiliary references to confirm the coordination of pyridine to unsaturated metal centers, further validating the presence of active Lewis acid sites responsible for substrate activation in the catalytic process [43,44,45]. The improved CO_2_ adsorption performance of the composite stems from its ability to concentrate CO_2_ near active sites, thereby accelerating cycloaddition reaction kinetics [46,47,48]. This synergistic enhancement of both acidity and adsorption capacity endows the HK@EPB catalyst with exceptional activity, resulting in superior CO_2_ conversion efficiency under identical reaction conditions [49,50,51,52].

**Figure 8:**
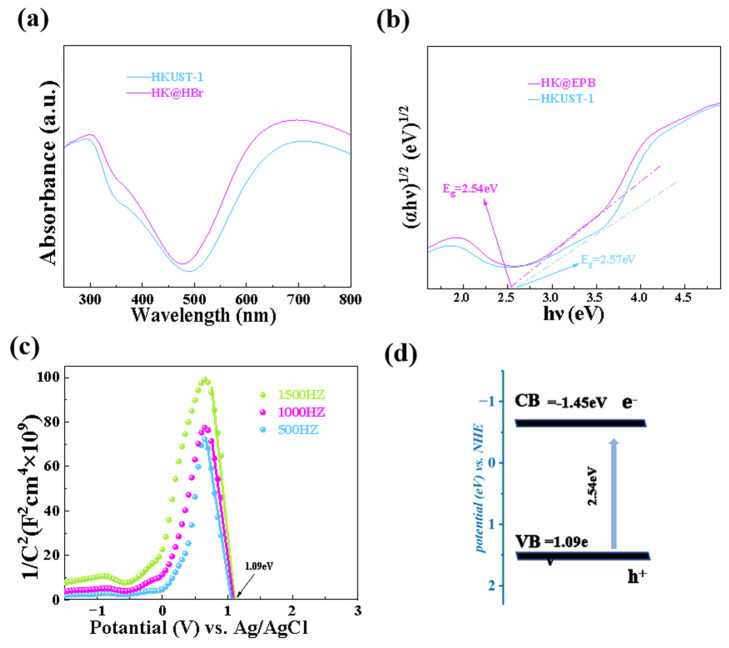
(**a**) UV-vis diffuse reflection spectroscopy of samples, (**b**) the corresponding Tauc plots of samples, (**c**) Mott–Schottky plots of HKUST-1 and HK@EPB, (**d**) Band structure diagram.

The effect of EPB immobilization on the light-harvesting properties of HKUST-1 was investigated using UV-vis diffuse reflectance spectroscopy (DRS). As shown in Figure 8a, compared with pristine HKUST-1, the EPB-immobilized composite did not exhibit significant changes in absorption intensity or spectral response range. EPB itself is a colorless and transparent substance and does not significantly enhance the UV-vis diffuse reflectance performance of the composite.

The flat-band potential determined by the Mott–Schottky curves in Figure 8c, combined with the bandgap width (which shows little change) calculated from Figure 8b, enables the construction of the band structure diagram in Figure 8d.

Although the bandgap shows little change, considering the (perhaps slightly enhanced) light absorption properties of the EPB-immobilized composite, it is suggested that the optimized EPB loading employed in this study contributes to superior photocatalytic performance under the experimental conditions. This is because even when the bandgap remains largely unchanged, any improvement in light utilization efficiency and suppression of electron–hole recombination can still enhance photocatalytic activity.

**Figure 9:**
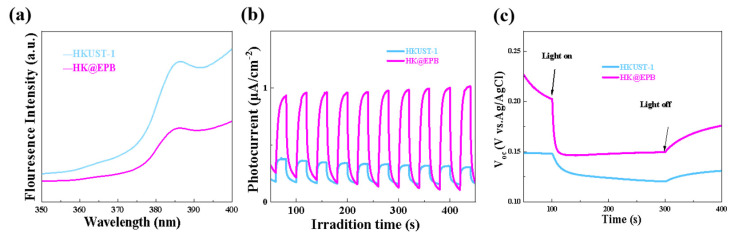
(**a**) Photoluminescence (PL) spectra (λex = 410 nm), (**b**) transient photocurrent response, and (**c**) open-circuit voltage (Voc) of HKUST-1 and HK@EPB.

Subsequent photoelectrochemical analysis of the prepared catalysts indicates that photoluminescence (PL) signals can serve as a proxy for the efficacy of separating photoinduced electron–hole pairs (Figure 9a). Notably, the EPB-immobilized sample exhibits a progressively diminished PL intensity compared with pristine HKUST-1, which is generally indicative of suppressed radiative recombination and more efficient charge carrier separation or interfacial transfer. This behavior suggests that the incorporation of EPB modulates the excited-state relaxation pathways of HKUST-1, favoring nonradiative charge utilization rather than direct electron–hole recombination.

Consistent with the PL results, transient photocurrent response measurements (Figure 9b) reveal a rapid rise and stable current density upon light irradiation, confirming the effective generation of photoexcited carriers under visible-light illumination. The prompt recovery of the current to its baseline upon light-off is attributed to carrier recombination in the absence of photoexcitation.

To further probe charge recombination dynamics, open-circuit voltage decay (OCVD) measurements were performed under steady-state illumination (Figure 9c). The OCVD profiles indicate extended minority carrier lifetimes for the composite, suggesting that the HK@EPB architecture establishes continuous electron transport pathways, thereby promoting charge separation and prolonging electron longevity. These results affirm the feasibility of visible-light-driven carrier generation (λ > 420 nm) in HK@EPB, highlighting its effectiveness in facilitating visible-light-assisted photothermal catalytic processes through improved charge separation and carrier lifetime.

**Figure 10:**
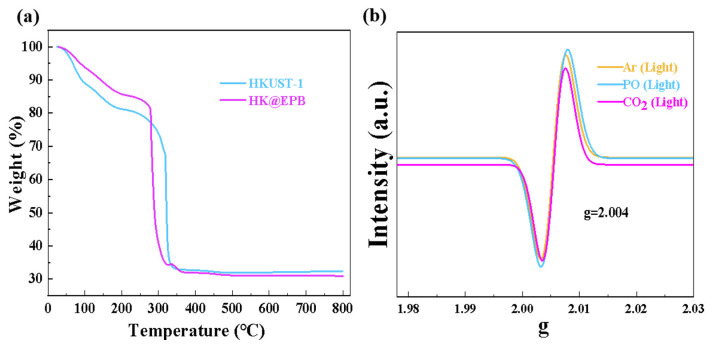
(**a**) Thermogravimetric plots of HKUST-1 and HK@EPB, (**b**) In situ EPR spectra of HK@EPB.

From Figure 10a, it can be observed that HKUST-1 exhibits a 10% weight loss before approximately 100 °C due to the desorption of adsorbed water or solvent, followed by the rapid decomposition of the MOF between 250–350 °C, resulting in a relatively low residual mass. In contrast, the HK@EPB composite material, after a similar initial weight loss, demonstrates a broader and more gradual decomposition range, with significantly improved thermal stability and a higher final residual mass. This indicates that the introduction of EPB effectively enhances the thermal stability and structural tolerance of the material.

In situ electron paramagnetic resonance (EPR) spectroscopy studies confirmed the activation of reaction substrates during the photofixation of CO_2_ on HK@EPB in Figure 10b. Consistent with previous findings, the Lorentzian signal at g = 2.004 was attributed to unpaired electrons on the carbon atoms of the catalyst. The attenuated EPR signal observed under a CO_2_ atmosphere indicate electron transfer from the catalyst to CO_2_, while no transfer to PO was detected, suggesting that PO is not activated via electron transfer. The persistently strong EPR signal observed in a PO atmosphere implies extremely limited electron transfer to PO.

**Figure 11:**
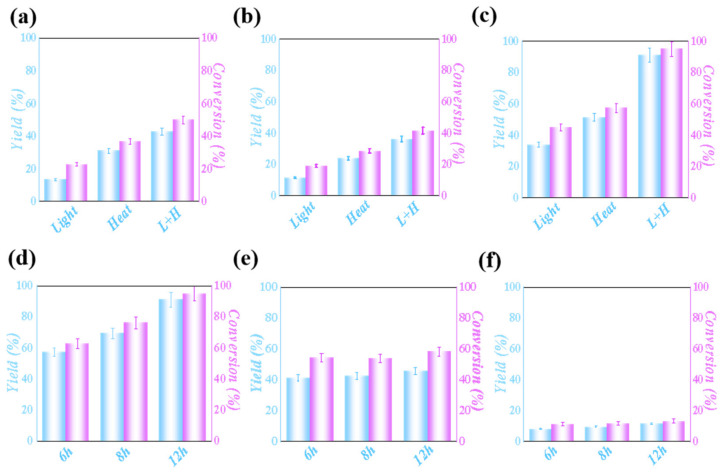
The catalytic performance of (**a**) EPB, (**b**) HKUST-1 and (**c**) HK@EPB under different conditions. Performance comparison of corresponding reaction times at different temperatures, (**d**) 80 °C, (**e**) 60 °C and (**f**) 25 °C. (The catalytic performance evaluation of the CO_2_ cycloaddition reaction was conducted with at least three parallel experiments, and the results are presented as ‘mean ± standard deviation’. An internal standard method was employed for gas chromatography quantitative analysis, with the relative error controlled within ±3%).

Systematic evaluation of EPB, HKUST-1, and HK@EPB catalysts was conducted under controlled conditions (1 MPa CO_2_ pressure, 0.07% mol TBAB) using three activation modes: photocatalysis, thermal catalysis, and photothermal synergistic catalysis. As shown in Figure 11a–c, at a constant temperature of 80 °C, both EPB and HKUST-1 exhibited progressively enhanced activity across the three activation modes (photocatalysis, thermal catalysis, and photothermal catalysis). The HK@EPB composite demonstrated superior performance, achieving 95% selectivity for the desired cyclic carbonate within 12 h under photothermal conditions. Importantly, the pronounced activity enhancement observed at elevated temperatures indicates that thermal input not only accelerates intrinsic reaction kinetics, but also facilitates mass transfer and reaction dynamics, thereby enhancing the overall efficiency of the light-assisted process. Moderate thermal energy promotes epoxide ring-opening dynamics, facilitates the diffusion of reaction intermediates within the HKUST-1 pores, and accelerates the desorption of cyclic carbonate products, thereby synergistically coupling with light-induced CO_2_ activation pathways. Further temperature-dependent experiments (Figure 11d–f) revealed a dramatic decline in catalytic activity at lower temperatures, with only marginal improvements even upon prolonged reaction times, underscoring the essential role of thermal assistance in this photothermal catalytic system.

**Figure 12:**
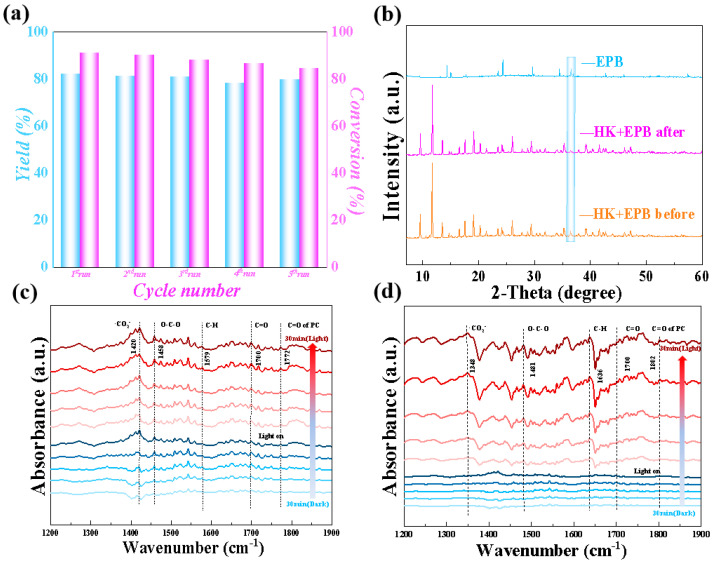
(**a**) Cyclic stability test of HK@EPB, (**b**) Post-cycling XRD analysis, (**c**) HKUST-1 and (**d**) HK@EPB In situ infrared images.

After five cycles, examination of the catalyst showed no significant decline in performance, as illustrated in Figure 12a. Analysis of the cycled catalyst using X-ray diffraction (XRD), shown in Figure 12b, confirmed the maintenance of structural integrity and the preservation of the ionic liquid species. These results robustly demonstrate the long-term stability of the EPB-immobilized composite material. This phenomenon can be attributed to the confinement of the EPB ionic liquid within the nanoscale pores and cage-like structures of HKUST-1 through physical adsorption or chemical interactions. This spatial confinement effect effectively prevents the leaching or volatilization-induced loss of EPB molecules—particularly the Br^−^ nucleophilic components—during use and recovery processes. This represents a fundamental distinction from homogeneous ionic liquids or TBAB co-catalysts.

In situ infrared spectroscopy was employed to monitor the dynamic changes in reactive ·CO_2_^−^-derived intermediates during the reaction. As shown in Figure 12c, minimal radical signals were detected in pristine HKUST-1 under dark conditions. Notably, the intensity of the ·CO_2_^−^ radical did not increase upon light exposure, indicating ineffective light-induced radical activation in the pure MOF. In contrast, the HKUST-1 composite immobilized with EPB exhibited significantly stronger ·CO_2_^−^ radical signals under light conditions compared to dark conditions, which is consistent with the in situ EPR results. This light-induced radical generation pattern aligns well with the superior cycloaddition performance of the composite under photothermal conditions.

Furthermore, the peak observed at 1700 cm^−1^ is attributed to the characteristic stretching vibration of the carbonate (C=O) species formed after the successful insertion of CO_2_ into the ring-opened epoxide intermediate. This assignment is in good agreement with the established reaction mechanism for CO_2_ cycloaddition to epoxides, which proceeds via a carbonate intermediate. Meanwhile, the characteristic vibration peak of carbonate at 1481 cm^−1^ is assigned to the asymmetric stretching vibration of O-C-O and the stretching vibration of C=O, also resulting from the formation of R-OCOO on the composite. Moreover, the gradually intensifying absorption peak at 1802 cm^−1^ corresponds to the C=O stretching vibration of cyclic carbonate, confirming the formation of propylene carbonate (PC).

**Figure 13:**
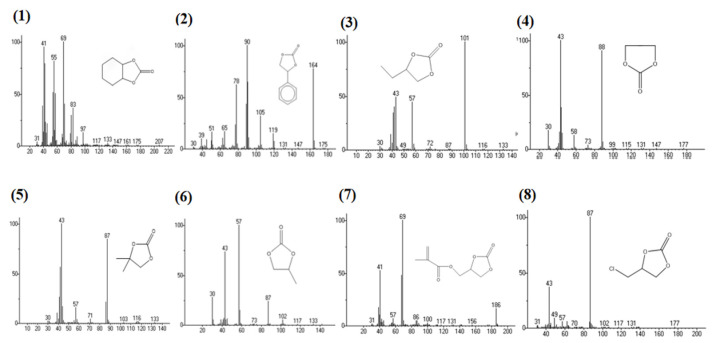
GC-MS analysis of (**1**) Hexahydro-1,3-benzodioxol-2-one, (**2**) 4-Phenyl-1,3-dioxolan-2-one, (**3**) 4-Propyl-1,3-dioxolan-2-one, (**4**) 1,3-Dioxolan-2-one, (**5**) 4,4-Dimethyl-1,3-dioxolan-2-one, (**6**) 4-Methyl-1,3-dioxolan-2-one, (**7**) 4-((2-Methylacryloyloxy) methyl)-1,3-dioxolan-2-one and (**8**) 4-(2-Chloroethyl)-1,3-dioxolan-2-one.

**Figure 14:**
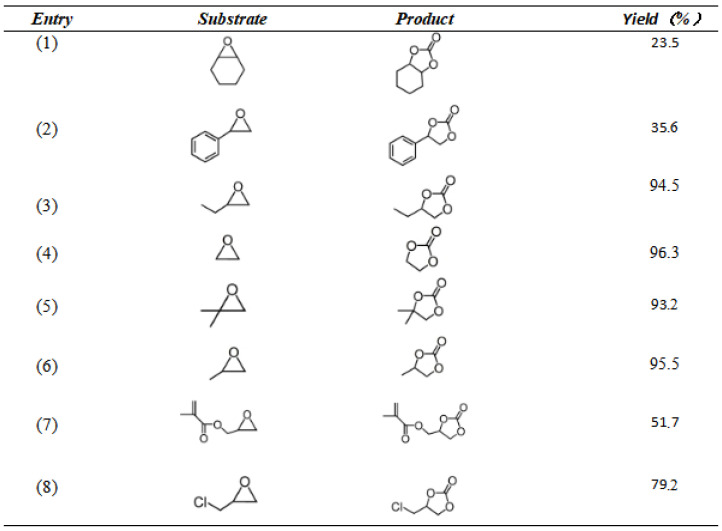
Conversion ability of HK@EPB to different epoxides. Reaction conditions: 4 mmol epoxide, 0.04 mmol co-catalyst, solvent CH_3_CN (1 mL), catalyst 40 mg, CO_2_ (1.0 MPa), 353 K, visible light (100 mW·cm^−2^, λ ≥ 420 nm), 10 h. The product yields were quantified by GC-FID with CFCl_3_ as an internal standard.

The GC-MS spectra of the products from the cycloaddition reaction of different epoxides in Figure 13 indicate that the composite catalyst can efficiently catalyze all the aforementioned epoxides to generate the corresponding cyclic carbonate products. The data in Figure 14 show that for epoxides with small R groups, such as ethylene oxide, propylene oxide, and other similar epoxides, the yield of the target cyclic carbonate exceeds 95% under 80 °C and 1 MPa. Specifically, the yields of ethylene oxide, propylene oxide, and methyl-substituted propylene oxide all exceed 90%. In contrast, epoxides bearing bulky R groups exhibit significantly lower yields, which is likely due to steric hindrance impeding the reaction process.

**Figure 15:**
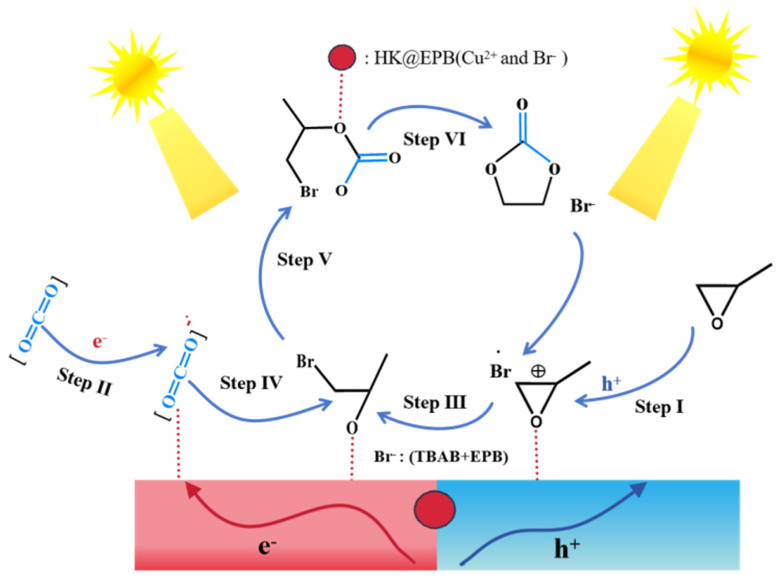
Diagram of the mechanism of photothermal catalytic reaction.

This study discusses a triple synergistic mechanism in the photothermal catalytic CO_2_ cycloaddition reaction using HK@EPB composites (as illustrated in Figure 15): (1) Under light irradiation, the semiconductor characteristics of HKUST-1 may enable valence band electrons to be excited into the conduction band, with the generated photoelectrons potentially contributing to the activation of CO_2_ into ·CO_2_^−^ radicals [53]. (2) Nucleophilic Br^−^ species derived from EPB and a small amount of TBAB, along with photogenerated bromine radicals [54], may synergistically promote epoxide ring-opening in cooperation with Lewis acidic Cu sites, thereby helping to lower the activation barrier for CO_2_ insertion [55]. (3) Meanwhile, the reversible Cu^2+^/Cu^+^ redox couple in HKUST-1 may act as an electron mediator under light irradiation [56,57], potentially facilitating the capture and transfer of photoexcited electrons and thereby supporting the formation and stabilization of ·CO_2_^−^ intermediates [58]. Thermal input may further enhance charge mobility and interfacial reaction kinetics, possibly leading to improved overall catalytic efficiency. This photo-assisted thermal synergistic mechanism could offer useful design principles for developing high-efficiency CO_2_ conversion catalysts.

## 4. Conclusions

In summary, this study successfully constructs an ionic liquid microenvironment catalytic system by integrating the ionic liquid EPB with the HKUST-1 metal–organic framework, enabling efficient CO_2_ cycloaddition under mild photothermal conditions (80 °C, 1 MPa). In this system, HKUST-1 provides abundant Lewis acid sites for effective CO_2_ adsorption and activation, while EPB and TBAB supply nucleophilic Br^−^ species to facilitate epoxide ring-opening, collectively establishing an acid-assisted nucleophilic ring-opening catalytic environment. Furthermore, the ionic liquid microenvironment created by EPB on the surface or within the pores of the MOF helps dissolve and concentrate both epoxides and CO_2_, improving mass transfer across the heterogeneous interface and promoting reactant contact. Under combined light irradiation and thermal input, the optimized composite achieves up to 95% selectivity within 12 h, outperforming pristine HKUST-1 and physically mixed counterparts. Beyond demonstrating an innovative strategy for constructing framework–ionic liquid cooperative active sites for designing multifunctional MOF catalysts, this work provides important methodological references for developing efficient CO_2_ conversion technologies under mild conditions. These findings offer valuable insights for advancing carbon neutrality goals.

## Figures and Tables

**Table 1:** Ion chromatography test data of HK@EPB.

Sample	Final Concentration (mg/kg)
HKUST-1	0
HK@EPB	1595.110

**Table 2:** Data on the specific surface area and pore size of the sample.

Sample	BET Surface Area (m^2^/g)	Pore Volume (cm^3^/g)
HKUST-1	1088	5.98
HK@EPB	558	2.40

## Data Availability

Data are contained within the article and Supplementary Materials.

## Supplementary Materials

The following supporting information can be downloaded at: https://www.mdpi.com/article/10.3390/molecules31132332/s1, Figure S1. GC-MS diagram of phenol capturing bromine radicals. Figure S2. Diagram of the photocatalytic reaction setup. Figure S3. Whether to add the TBAB reaction performance diagram. Figure S4. HK@EPB Ion chromatogram. Table S1. Lewis acid site (Newly tested samples). Table S2. Lewis acid site (previously tested samples). Table S3. Performance comparison of reported CO_2_ cycloaddition MOF catalysts. Table S4. (ICP-OES). References [59,60,61,62,63,64,65,66,67] are cited in the Supplementary Materials.

## References

[1] Wu Z., Ge Q., Cong H., Jiang N., Zhao W., Yang S. (2025). Ligand-regulated oxygen vacancies in Ti-MOFs for visible-light-driven CO_2_ cycloaddition to cyclic carbonates. Sep. Purif. Technol..

[2] Zhang S., Lin Y., Wang Q., Yang J., Yang Z., Chen X., Wang J., Xiao J. (2025). Heat treatment of Fe-doped Zr-MOF catalysts for dual Lewis acid sites promoted CO_2_ cycloaddition. ACS Appl. Nano Mater..

[3] Li M., Zhang J., Wei Z., Gao Y., Zhao Y., Teng Y., Wang H., Zhang R., Yang Y. (2025). NH_2_-MIL-101(Fe) nanocrystals synthesized by the ionic liquid-ethanol interface for efficient CO_2_ fixation at mild conditions. ACS Appl. Mater. Interfaces.

[4] Gao C., Wu Y., Ding L., Xu N., Li Y., Hai W., Bai J., Liu J., Yang Y. (2025). A highly stable zinc phosphonate for efficient heterogeneous catalytic CO_2_ cycloaddition and ring-opening reaction. J. Environ. Chem. Eng..

[5] Gao C., Ding L., Li Y., Xu N., Wu Y., Wang W., Liu J., Yang Y. (2025). Anchoring Ag(I) into MOF-253 for effectively catalyzing cycloaddition of CO_2_ with alkynyl alcohols/amine under ambient conditions. Inorg. Chem..

[6] Decortes A., Belmonte M., Benet-Buchholz J., Kleij A. (2010). Efficient carbonate synthesis under mild conditions through cycloaddition of carbon dioxide to oxiranes using a Zn(salphen) catalyst. Chem. Commun..

[7] Dai W., Luo S., Yin S., Au C. (2009). The direct transformation of carbon dioxide to organic carbonates over heterogeneous catalysts. Appl. Catal. A.

[8] Sun J., Han L., Cheng W., Wang J., Zhang X., Zhang S. (2011). Efficient acid-base bifunctional catalysts for the fixation of CO_2_ with epoxides under metal and solvent-free conditions. ChemSusChem.

[9] Zalomaeva O., Chibiryaev A., Kovalenko K., Kholdeeva O., Balzhinimaev B., Fedin V. (2013). Cyclic carbonates synthesis from epoxides and CO_2_ over metal-organic framework Cr-MIL-101. J. Catal..

[10] Zhao Y., Yao C., Chen G., Yuan Q. (2013). Highly efficient synthesis of cyclic carbonate with CO_2_ catalyzed by ionic liquid in a microreactor. Green Chem..

[11] Alsina J., Chiva C., Giralt E., Albericio F., Rabanal F. (1998). Active carbonate resins: Application to the solid-phase synthesis of alcohol, carbamate and cyclic peptides. Tetrahedron.

[12] Fearn J. (2015). Development, optimization and application of catalysts in cyclic carbonate synthesis and olefin epoxidation. Der Anaesthesist.

[13] Beyzawi M., Stephenson C., Liu Y., Karagiaridi O., Hupp J., Farha O. (2015). Metal-organic framework-based catalysts: Chemical fixation of CO_2_ with epoxides leading to cyclic organic carbonates. Front. Energy Res..

[14] Borah R., Deori N., Lahkar S., Paul S., Brahma S. (2024). Carbon dioxide cycloaddition to epoxides: A comparative study on the catalytic activities of two binary catalysts V^IV^O(N_2_O_2_)/TBAB and V^V^O_2_(ON)/TBAB and in situ derivation of a bifunctional catalyst. Catal. Lett..

[15] Wang B., Cao X., Wang L., Meng X., Wang Y., Sun W. (2024). Co(II)-N_4_ catalysts for the coupling of CO_2_ with epoxides into cyclic carbonates: Catalytic activity, computational and kinetic studies. Inorg. Chem..

[16] Perez-Sena W., Ciccarelli F., Ernen K., Di Serio M., Russo V., Salmi T. (2025). A pathway to cyclic carbonates: Cycloaddition of carbon dioxide to epoxidized methyl oleate on grafted heterogeneous catalysts. J. CO_2_ Util..

[17] Yang J., Sun X. (2024). Nitrogen-doped carbon dots as acid-base bifunctional and efficient catalysts for the cycloaddition of CO_2_ with epoxides. New J. Chem..

[18] Campisciano V., Valentino L., Morena A., Santiago-Portillo A., Saladino N., Gruttadauria M., Aprile C., Giacalone F. (2022). Carbon nanotube supported aluminum porphyrin-imidazolium bromide crosslinked copolymer: A synergistic bifunctional catalyst for CO_2_ conversion. J. CO_2_ Util..

[19] Yu H., Shang F., Chu Q., Wang P., Wang M., Zhu H., Song F., Yang H., Diao T. (2020). Cleaner and atomic economy production of hydroxylamine hydrochloride under solvent-free conditions through process intensification. J. Clean. Prod..

[20] Zhang X., Wang J., Bian Y., Lv H., Qiu B., Zhang Y., Qin R., Zhu D., Zhang S., Li D. (2022). A novel conjugated microporous polymer microspheres comprising cobalt porphyrins for efficient catalytic CO_2_ cycloaddition under ambient conditions. J. CO_2_ Util..

[21] Sarkar D., Weetman C., Dutta S., Schubert E., Jandl C., Koley D., Inoue S. (2020). N-Heterocyclic carbene-stabilized germa-acylium ion: Reactivity and utility in catalytic CO_2_ functionalizations. J. Am. Chem. Soc..

[22] Johnson M., Rogers D., Kaminsky W., Cossairt B. (2021). CO_2_ hydrogenation catalyzed by a ruthenium protic N-heterocyclic carbene complex. Inorg. Chem..

[23] Wang G., Ren S., Zhang J., Ning X., Liang W., Zhang N., Wang C. (2020). Influence mechanism of alkali metals on CO_2_ gasification properties of metallurgical coke. Chem. Eng. J..

[24] Endrődi B., Samu A., Kecsenovity E., Halmágyi T., Sebök D., Janáky C. (2021). Operando cathode activation with alkali metal cations for high current density operation of water-fed zero-gap carbon dioxide electrolysers. Nat. Energy.

[25] Zhao S., Wang K., Yang B., Sun Z., Zhao Y. (2025). Synergistic catalysis of dual-sites promoted cycloaddition of CO_2_ with epoxides. Fuel.

[26] Wang L., Feng Z., Hou Q., Dang Z., Yu Y., Yang C., Tang B., Zhou Q., Hua X., Wei R. (2025). Quaternary ammonium salt functionalized copper phthalocyanine-graphene oxide hybrids for cocatalyst-free carbon dioxide cycloaddition. Adv. Compos. Hybrid Mater..

[27] Nataj S., Kaliaguine S., Fontaine F. (2025). CO_2_ fixation reaction over pyrimidinium-based dicationic ionic liquid in MIL-101(Cr). Appl. Catal. A.

[28] Natongchai W., Crespy D., D’Elia V. (2025). CO_2_ fixation: Cycloaddition of CO_2_ to epoxides using practical metal-free recyclable catalysts. Chem. Commun..

[29] Honores J., Quezada D., Camarada M., Ramirez G., Isaacs M. (2005). Integrated experimental and theoretical insights into CO_2_ fixation: Tetraazamacrocyclic catalysts in ionic liquids for cyclic carbonate formation. RSC Sustain..

[30] Sahil, Gupta N. (2024). Cyclic carbonates: Treasure of fine chemicals obtained from waste stream CO_2_ over carbon-based heterogeneous catalysts. Renew. Sustain. Energy Rev..

[31] Huang Y., Fan L., Cai Z., Zhang L., Huang K. (2023). Zinc-supported nitrogen-rich carbon catalyst for efficient carbon dioxide cycloaddition with epoxides under solvent-free conditions. Asian J. Org. Chem..

[32] Li W., Wang S., Huang Y., Fan L., Zhang L., Huang K. (2024). Anchoring bimetal zinc-aluminum sites on nitrogen-doped carbon catalyst for efficient conversion of CO_2_ into cyclic carbonates. ChemistrySelect.

[33] Tapiador J., García-Rojas E., Leo P., Martos C., Calleja G., Orcajo G. (2023). Copper MOFs performance in the cycloaddition reaction of CO_2_ and epoxides. Microporous Mesoporous Mater..

[34] Amini M., Yousofvand A., Hosseinifard M., Bayrami A., Janczak J. (2024). Synthesis and characterization of a new copper-based polyoxomolybdate and its catalytic activity for azide-alkyne cycloaddition reaction under UV light irradiation. Sci. Rep..

[35] Ye S., Wang F., Zhang L., Fan F., Zhang X., Wang T., Fu Y. (2025). Polymer brush-grafted metal-organic framework nanoplates for enhanced catalysis of CO_2_ cycloaddition with epoxides. Inorg. Chem..

[36] Jan R., Ghosh T.K., Shaikh R.R., Bhagavathy S., Shakeela K., Rao G.R. (2024). Catalytic coupling of CO_2_ and epoxides with metal substituted Keggin based hybrid materials. J. Organomet. Chem..

[37] Gourkhede R., Balakrishna M. (2025). Iminophosphorano-phosphine based Cu^I^ complexes: Utility in 1,3-dipolar azide-alkyne cycloaddition and A^3^-coupling reaction. New J. Chem..

[38] Roland G., Hurdal K., Bouchouicha A., Dowe N., Davis R., Collins S. (2024). [2+2] Photocycloadditions to form cyclobutanes and bicyclo[2.1.1]hexanes employing copper-based photocatalysis. ACS Catal..

[39] Singh R., Singh G., George N., Singh G., Dalal A., Singh H., Kaur G., Singh J. (2025). Chalcone-ensembled 1,2,3-triazole via click chemistry: Selective ‘Turn-On’ detection of Cu(II) ions via photoinduced electron transfer in real water samples and computational analysis. J. Mol. Struct..

[40] Rana S., Cho J. (2010). Functionalization of carbon nanotubes via Cu(I)-catalyzed Huisgen [3+2] cycloaddition “click chemistry”. Nanoscale.

[41] Singh R., Singh G., George N., Singh G., Gupta S., Singh H., Kaur G., Singh J. (2023). Copper-based metal-organic frameworks (MOFs) as an emerging catalytic framework for click chemistry. Catalysts.

[42] Kurisingal J.F., Rachuri Y., Gu Y., Kim G., Park D. (2019). Binary metal-organic frameworks: Catalysts for the efficient solvent-free CO_2_ fixation reaction via cyclic carbonates synthesis. Appl. Catal. A.

[43] Jeong G., Kathalikkattil A., Babu R., Chung Y., Park D. (2018). Cycloaddition of CO_2_ with epoxides by using an amino-acid-based Cu(II)-tryptophan MOF catalyst. Catal. Today.

[44] Fan Y., Shan H., Wu Y., Zhu H. (2025). Ionic liquids-based ionic metal-organic frameworks (MOFs): A single catalyst with dual active centers for the cycloaddition of carbon dioxide. Appl. Catal. A.

[45] Bao W., Kuai J., Gao H., Zheng M., Sun Z., He M., Chen Q., Zhang Z. (2024). Ionic liquid post-modified carboxylate-rich MOFs for efficient catalytic CO_2_ cycloaddition under solvent-free conditions. Dalton Trans..

[46] Wang H., Gao Z., Sun J. (2024). The synthesis of amide bond by a simple one-step method to connect ionic liquid and MIL-101-NH_2_ firmly for efficient CO_2_ cycloaddition. Sep. Purif. Technol..

[47] Niu J., Zhang D., Gao X., Gao Y., Wu J., Han L., Zhu N. (2024). Ionic liquid immobilized Fe-MIL-101-NH_2_ efficiently catalyzes the chemical fixation of CO_2_ with epoxides to form cyclic carbonates. ChemistrySelect.

[48] Pei X., Song W., Zhang Z., Zhao Y., Li Z. (2023). CO_2_-responsive CuI-ionic liquid/zeolitic imidazolate frameworks stabilized Pickering emulsions for azide-alkyne click reaction. ACS Appl. Nano Mater..

[49] Sun W., Ran W., Guo L., Song X., Lu D. (2023). Preparation of ionic liquids immobilized on FMIL-101 catalysts for conversion of CO_2_ to propylene carbonate. China Pet. Process. Petrochem. Technol..

[50] Wang X., Wang Z., Gao S. (2007). A pillared layer MOF with anion-tunable magnetic properties and photochemical [2+2] cycloaddition. Chem. Commun..

[51] Zhang Y., Chen C., Cai L., Tan B., Yang X.D., Zhang J., Ji M. (2017). Post-cycloaddition modification of a porous MOF for improved GC separation of ethanol and water. Dalton Trans..

[52] Wang T., Song X., Xu H., Chen M., Zhang J., Ji M. (2021). Recyclable and magnetically functionalized metal-organic framework catalyst: IL/Fe_3_O_4_@HKUST-1 for the cycloaddition reaction of CO_2_ with epoxides. ACS Appl. Mater. Interfaces.

[53] Kathalikkattil A., Gu Y., Kurisingal J., Lee H., Kim H., Choe Y., Park D.W. (2021). A catalytic approach of blending CO_2_-activating MOF struts for cycloaddition reaction in a helically interlaced Cu(II) amino acid imidazolate framework: DFT-corroborated investigation. Res. Chem. Intermed..

[54] Wang Y., Wu Y., Xie J., Ge H., Hu X. (2013). Multi-walled carbon nanotubes and metal-organic framework nanocomposites as novel hybrid electrode materials for the determination of nano-molar levels of lead in a lab-on-valve format. Analyst.

[55] Wu Y., Zhang C., Su X., Shi S., Liu P., Oderinde O., Xiao G., Zhang Y. (2025). Catalytic conversion of carbon dioxide into cyclic carbonates and fuels over metal ionic liquid complexes: Experimental and DFT studies. Energy.

[56] Li J., Huang R., Chen L., Xia Y., Yan G., Liang R. (2023). Mixed valence copper oxide composites derived from metal-organic frameworks for efficient visible light fuel denitrification. RSC Adv..

[57] Xie G., Qiu J., Li H., Li S., Zeng Y., Zheng K., Wang X. (2025). Facile construction of heterogeneous dual-ionic poly(ionic liquid)s for efficient and mild conversion of CO_2_ into cyclic carbonates. J. Environ. Sci..

[58] Adegoke K., Akpomie K., Okeke E., Olisah C., Malloum A., Maxakato N., Ighalo J., Conradie J., Ohoro C., Amaku J. (2024). UiO-66-based metal-organic frameworks for CO_2_ catalytic conversion, adsorption and separation. Sep. Purif. Technol..

[59] Liang R., Zhang C., Wang Y., Wu L., Zhao Y., Liang S., Yang G., Long J. (2026). Efffcient Halogen Radical-Mediated Photosynthesis of Cyclic Carbonates over Perylene Diimide-Grafted Zirconium Metal−Organic Frameworks with Visible Light Irradiation. J. Am. Chem. Soc..

[60] Adegoke K., Maxakato N. (2021). Porous metal-organic framework (MOF)-based and MOF-derived electrocatalytic materials for energy conversion. Mater. Today Energy.

[61] Lu G., Zhang P., Sun D., Wang L., Zhou K., Wang Z., Guo G. (2014). Gold catalyzed hydrogenations of small imines and nitriles: Enhanced reactivity of Au surface toward H_2_ via collaboration with a Lewis base. Chem. Sci..

[62] Radwan A., Jin H., He D., Mu S. (2021). Design engineering, synthesis protocols, and energy applications of MOF-derived electrocatalysts. Nano-Micro Lett..

[63] Qiu J., Zhang X., Feng Y., Zhang X., Wang H., Yao J. (2018). Modified metal-organic frameworks as photocatalysts. Appl. Catal. B.

[64] Billo T., Shown I., Anbalagan A., Effendi T., Sabbah A., Fu F., Chu C., Woon W., Chen R., Lee C. (2020). A mechanistic study of molecular CO_2_ interaction and adsorption on carbon implanted SnS_2_ thin film for photocatalytic CO_2_ reduction activity. Nano Energy.

[65] Mu Q., Zhu W., Li X., Zhang C., Su Y., Lian Y., Qi P., Deng Z., Zhang D., Wang S. (2020). Electrostatic charge transfer for boosting the photocatalytic CO_2_ reduction on metal centers of 2D MOF/rGO heterostructure. Appl. Catal. B.

[66] Wei S., Heng Q., Wu Y., Chen W., Li X., Shangguan W. (2021). Improved photocatalytic CO_2_ conversion efficiency on Ag loaded porous Ta_2_O_5_. Appl. Surf. Sci..

[67] Elhenawy S., Khraisheh M., AlMomani F., Walker G. (2020). Metal-organic frameworks as a platform for CO_2_ capture and chemical processes: Adsorption, membrane separation, catalytic-conversion, and electrochemical reduction of CO_2_. Catalysts.

