# Supplementary material for: Ionic Liquid Microenvironment Engineering in HKUST-1 for Efficient Photothermal CO2 Cycloaddition
Source: Molecules. 2026 Jul 3;31(13):2332. doi: 10.3390/molecules31132332 (PMC13363707; doi:10.3390/molecules31132332)
Supplement: Supplementary file 1 [file molecules-31-02332-s001.zip › molecules-4370368-supplementary.pdf]

Ionic Liquid Microenvironment Engineering in HKUST-1 for Efficient Photothermal CO_2 Cycloaddition

Renkun Huang [1,2], Haohao Yan [1,3], Runling Huang [1,3], Chen Zhou [1], Qiuzhong Li [1,*], Lu Chen [1] and Ruowen Liang [1,*]

[1] Province University Key Laboratory of Green Energy and Environment Catalysis, Ningde Normal University, Ningde 352100, China; t1432@ndnu.edu.cn (R.H.); y15038159528@163.com (H.Y.); 17341976356@163.com (R.H.); zdfhju@163.com (C.Z.); chenlu199104@163.com (L.C.)

[2] Fujian Provincial Key Laboratory of Featured Materials in Biochemical Industry, Ningde Normal University, Ningde 352100, China

[3] State Key Laboratory of Photocatalysis on Energy and Environment, Fuzhou University, Fuzhou 350002, China

Experimental

GC-FID analysis

A Shimazu gas chromatography (GC-2014) with R-R-smz-C1 autosampler (Shimazu, AOC-20i) was used. Capillary columns (SH-Polar Wax) were used for all injections. Hydrogen was the carrier gas (25 mL/min) and the Column temperature control program was: 60 °C initial temperature held for 13.5 min, then 10 °C/min to 170 °C, then 35 °C/min to 250 °C and held for 2.5 min. Detection was by an FID (250 °C, hydrogen flow 3.2 mL/min, air flow 25.5 mL/min, nitrogen makeup flow 25 mL/min). Analysis of data was performed using OpenLab CDS Chemstation. Calibration curves were generated in the software using a weighted linear regression.

GC-MS analysis

The temperature program for GC-MS analysis was set as follows: initial temperature at 50 °C for 1 min, then raised to 150 °C at 20 °C/min and held for 2 min, finally raised to 250 ℃ at 10 °C/min and held for 20 min. split ratio 1:1, split flow 5.0 mL/min, purge flow 5.0 mL/min, front intel option constant flow 1.5 mL/min, pressure 2 psi. Before injection, we pre-treated the samples: the reaction solution samples collected after the activity test were diluted 1000 times with acetonitrile as the solvent. The samples for the bromine radical capture experiment were injected at their original concentration.

PC generation under different wavelengths of light

The photocatalytic test was measured by using a Xe lamp (Beijing China Education Au-light Co., Ltd., CEL-HXF300-T3) with different band-pass filters (λ = 350±10, 400±10, 450±10, 500±10, 550±10, 600±10, 650±10 and 700±10 nm).

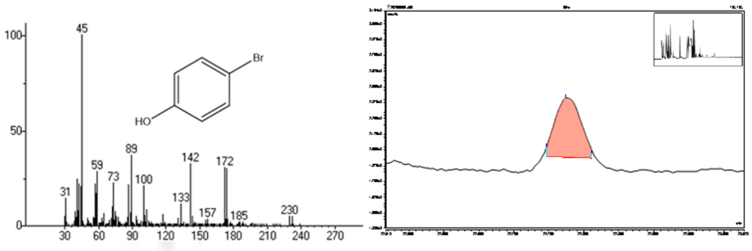

Figure S1. GC-MS diagram of phenol capturing bromine radicals.

NOTE: To confirm the presence of bromine radicals, phenol was employed as a scavenger to capture the signal of bromine radicals generated upon exposure to light. Analysis using GC-MS of the resulting product indicated the formation of tetrabromobophenol, as shown in Figure S1, providing evidence for the production of bromine radicals in the reaction[59].

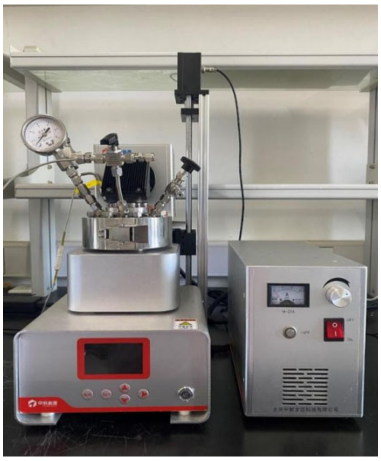

Figure S2. Diagram of the photocatalytic reaction setup.

NOTE: Details of the photocatalytic reactor operation: A quartz optical window was positioned directly above the reactor, with the Xe lamp light source horizontally aligned with the window for external irradiation. This setup enabled effective photocatalytic reactions while maintaining high-pressure sealing.

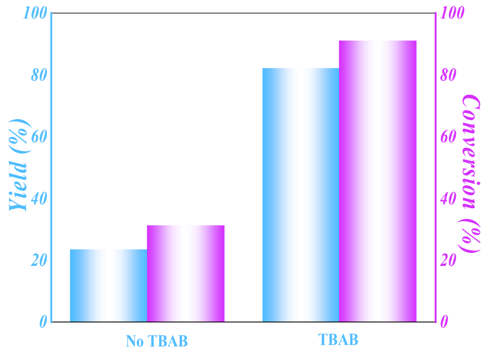

Figure S3. Whether to add the TBAB reaction performance diagram.

NOTE: As can be seen from Figure S3, at a temperature of 80 degrees Celsius and for a reaction duration of 12 hours, a comparison of reaction performance shows that the addition of a small amount of TBAB significantly enhances the reaction efficiency.

Table S1. Lewis acid site (Newly tested samples).

Sample	*Lewis acid sites (umol • g^{-1})*
HKUST-1	39.74
HK@EPB	56.57

Table S2. Lewis acid site (previously tested samples).

Sample	*Lewis acid sites (umol • g^{-1})*
HKUST-1	39.51
HK@EPB	57.48

NOTE: We conducted repeated pyridine-infrared tests on the other two samples. The results showed that the Lewis acid content of HKUST-1 was 39.51 $\mu mol{\cdot}g^{-1}$ (compared with 39.74 $\mu mol{\cdot}g^{-1}$ in the original manuscript), while that of HK@EPB was 57.48 $\mu mol{\cdot}g^{-1}$ (compared with 56.57 $\mu mol{\cdot}g^{-1}$ in the original manuscript). During the process of loading the ionic liquid, after multiple centrifugation and cleaning steps, the cleaning process itself may expose more metal sites. Perhaps the ionic liquid itself does not have Lewis acidity, but in the process of preparing this composite material, the acidity sites are inevitably increased, which can also explain that the increase in acidic sites is indeed limited.

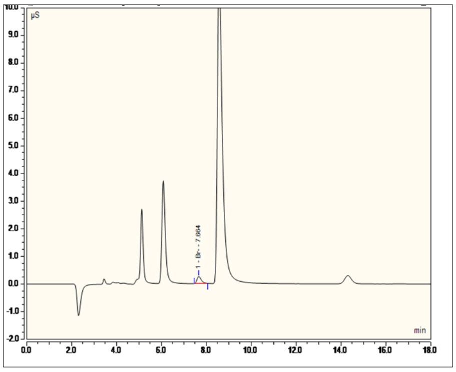

Figure S4. HK@EPB Ion chromatogram.

(Test item: Ion Chromatography (IC)

Instrument model: Thermo Scientific ICS-6000, USA)

NOTE: To quantitatively describe the immobilization amount of the ionic liquid in the composite, we performed ion chromatography to measure the bromine element in the composite, and the results are shown in the figure.

Table S3. Performance comparison of reported CO_2 cycloaddition MOF catalysts.

Entry	*Catalyst*	*Co-catalyst*	*Temperature*	*Pressure*	*Light source*	*Reaction rate ($mmol \cdot g^{-1} \cdot h^{-1}$)*	*Ref.*
1	HKUST-1@EPB	TBAB	353K	1Mpa	100 $mW \cdot cm^{-2}$ visible light ($\lambda \geq 420$ nm)	**68.4**	**This work**
2	UiO-67-B	TBAB	298 K	1 bar	Xenon lamp (300 W 18 A)	3.72	60
3	$FeNbO_4$/ NH_2-MIL125(Ti)	TBAB	348 K	1 bar	500 W visible light halogen lamp	0.2	61
4	Ce-BDC-NH_2	TBAB	298 K	1 bar	125 W medium-pressure mercury vapor lamp	0.12	62
5	$BiNbO_4$/ NH_2-MIL125(Ti)	TBAB	353 K	1 bar	300 W halogen lamp	0.062	63
6	Bi-PCN-224	TBAB	298 K	1 bar	300 W xenon lamp (full spectrum of light)	24.75	63
7	TpPa-1	TBAB	298 K	1 bar	455 nm blue LED	4.3	64

8	Mn-MOF	TBAB	353 K	0.1 MPa	300 W xenon lamp	5.95	65
9	IHEP-9	TBAB	298 K	0.1 MPa	45 W, 6500 K compact fluorescent lamp	1.67	66
10	UiO-bpydc(Zn)	TBAB	298 K	1 bar	300 W xenon lamp (18 A)	8.33	67

Table S4 (ICP-OES)

Basic information of the experiment		
Order Number	26030968632	
Testing items	Cu	
Sample quantity	2	
Testing date	2026/03/11	
Instrument model	ICP-OES:Agilent 5800	
Instrument parameters	RF Power：1200W	
	Plsama flow：12 L/min	
	Auxiliary flow： 1.0 L/min	
	Nebulizer flow：0.7 L/min	
	Sample uptake delay：30S	
Data Calculation Explanation	$C_X(mg/kg) = \frac{C_0(mg/L) * f * V_0(mL) * 10^{-3}}{m_0(g) * 10^{-3}} = \frac{C_1(mg/L) * V_0(mL) * 10^{-3}}{m_0(g) * 10^{-3}}$	(1)
	$W(\%) = \frac{C_X(mg/kg)}{10^6} * 100\%$	(2)

	$$C_1(mg/L) = C_0(mg/L) * f$$	(3)
	In the formula:	
	m_0： When analyzing the samples, the mass of the samples taken, expressed in grams (g), was recorded by the analytical balance and is presented in the B column of the following table.	
	V_0： After sample digestion, the volume of the solution after dilution, expressed in milliliters (mL), corresponds to the data in column C of the following table.	
	f: The dilution factor corresponds to the data in column F of the following table.	
	C_0： Test the concentration of the solution's elements, expressed in milligrams per liter (mg/L). This data was obtained through instrument testing and corresponds to the data in column E of the following table.	
	C_x: The final test results of the measured elements, expressed in units of milligrams per kilogram (mg/kg), are calculated according to the above formula (1) and correspond to the data in column H of the following table.	
	W(%): The final test results of the measured elements are expressed as percentages and are calculated according to the above formula (2), corresponding to the data in column I of the following table.	
	Note: For powder solid samples or other samples that require pre-treatment before digestion, the final results are calculated using formula (1) or (2). For liquid samples that can be directly tested, the final results are calculated using formula (3).	
Feedback on the problem situation:		

Test data

Sample Number	Sampling Quantity (g)	Total Volume V0 (mL	Test Element	Element Concentration C0 (mg/L) of Test Solution	Dilution factor f	Decomposition solution / original sample solution element concentration C1 (mg/L)	Element content of sample Cx (mg/kg)	Element content of sample W (%)
Before the reaction	0.501	10	Cu	6.4156	10.0000	64.1560	1280.5589	0.1281
	0.501	10	Cu	6.3906	10.0000	63.9060	1275.5689	0.1276
	0.501	10	Cu	6.3833	10.0000	63.8330	1274.1118	0.1274
After the reaction	0.5206	10	Cu	6.4566	10.0000	64.5660	1240.2228	0.1240
	0.5206	10	Cu	6.4387	10.0000	64.3870	1236.7845	0.1237
	0.5206	10	Cu	6.4289	10.0000	64.2890	1234.9020	0.1235

NOTE: Table S1 demonstrates that during the cyclic testing of the samples, the integrity of the samples was maintained and the copper content remained largely unchanged.

Reference

59. Liang, R.; Zhang, C.; Wang, Y.; Wu, L.; Zhao, Y.; Liang, S.; Yang, G.; Long, J. Efffcient Halogen Radical-Mediated Photosynthesis of Cyclic Carbonates over Perylene Diimide-Grafted Zirconium Metal−Organic Frameworks with Visible Light Irradiation. *J. Am. Chem. Soc.* **2026**, *148*, 6167-6177.

60. Adegoke, K.; Maxakato, N. Porous metal-organic framework (MOF)-based and MOF-derived electrocatalytic materials for energy conversion. *Mater. Today Energy* **2021**, *21*, 100816.

61. Lu, G.; Zhang, P.; Sun, D.; Wang, L.; Zhou, K.; Wang, Z.; Guo, G. Gold catalyzed hydrogenations of small imines and nitriles: enhanced reactivity of Au surface toward H_2 via collaboration with a Lewis base. *Chem. Sci.* **2014**, *5*, 1082-1090.

62. Radwan, A.; Jin, H.; He, D.; Mu, S. Design engineering, synthesis protocols, and energy applications of MOF-derived electrocatalysts. *Nano-Micro Lett.* **2021**, *13*, 132.

63. Qiu, J.; Zhang, X.; Feng, Y.; Zhang, X.; Wang, H.; Yao, J. Modified metal-organic frameworks as photocatalysts. *Appl. Catal. B* **2018**, *231*, 317-342.

64 .Billo, T.; Shown, I.; Anbalagan, A; Effendi, T.; Sabbah, A.; Fu, F.; Chu, C.; Woon, W.; Chen, R.; Lee, C.; et al. A mechanistic study of molecular CO_2 interaction and adsorption on carbon implanted SnS_2 thin film for photocatalytic CO_2 reduction activity. *Nano Energy* **2020**, *72*, 104717.

65. Mu, Q.; Zhu, W.; Li, X.; Zhang, C.; Su, Y.; Lian, Y.; Qi, P.; Deng, Z.; Zhang, D.; Wang, S.; et al. Electrostatic charge transfer for boosting the photocatalytic CO_2 reduction on metal centers of 2D MOF/rGO heterostructure. *Appl. Catal. B* **2020**, *262*, 118144.

66. Wei, S.; Heng, Q.; Wu, Y.; Chen, W.; Li, X.; Shangguan, W. Improved photocatalytic CO_2 conversion efficiency on Ag loaded porous Ta_2O_5. *Appl. Surf. Sci.* **2021**, *563*, 150273.

67. Elhenawy, S.; Khraisheh, M.; AlMomani, F.; Walker, G. Metal-organic frameworks as a platform for CO_2 capture and chemical processes: Adsorption, membrane separation, catalytic-conversion, and electrochemical reduction of CO_2. *Catalysts* **2020**, *10*, 1293.
